# Accountable Action End Points for Evaluating Artificial Intelligence in Digital Public Health

**DOI:** 10.2196/106243

**Published:** 2026-09-23

**Authors:** Xiang Zhou, Hongyan Liu, Zhengdong Hua

**Affiliations:** 1 Department of Cardiac Surgery, Wuhan Asia Heart Hospital, Wuhan University of Science and Technology, 753 Jinghan Avenue, Jianghan District, Wuhan, Hubei, 430022, China, 86 18707101850; 2 Wuhan Clinical Center for Cardiomyopathy, Wuhan, Hubei, China

**Keywords:** artificial intelligence, AI, public health, digital health, digital public health, implementation science, health equity, surveillance, algorithmic accountability

## Abstract

AI is increasingly embedded in digital public health systems, but model performance, system deployment, message delivery, alert volume, and user engagement do not establish whether an AI-supported workflow has changed public health practice. This viewpoint proposes a public health action end point—a prespecified, auditable, AI output–specific, actor-bound, time-bound, denominator-based way to specify and measure an existing proximal process or implementation end point. It begins with a defined AI output and records the corresponding action opportunity, accountable actor, action status, and evidence concerning the AI’s role in the action. A complete specification includes rules for repeated outputs, completed action, justified nonaction, missed action, unresolved cases, denominator loss, and paired safety, workload, privacy, equity, and model drift monitoring. A public health action end point is not a new causal outcome, reporting guideline, or validated surrogate for a population health benefit. An observed action rate alone does not establish that AI initiated or caused the action; causal claims require a documented attribution strategy and an appropriate comparator or causal design. The framework applies to research and production deployments but complements rather than replaces ethics review, trial registration, and jurisdiction-specific regulatory and governance obligations. It is intended to help authors, implementers, reviewers, and editors align claims with what an evaluation has actually measured.

## Use Cases Are Not Action End Points

Digital public health AI has moved rapidly from technical promise to implementation planning. Systems have been proposed for outbreak detection, syndromic surveillance, vaccination outreach, environmental risk warning, chronic disease registries, service triage, public communication, and administrative prioritization [1-4]. International guidance appropriately emphasizes transparency, accountability, safety, privacy, and equity [1,2].

These systems produce different kinds of outputs. A thresholded alert may directly create an action opportunity, a prioritization score or recommendation usually requires professional judgment before it does, and generated content or chatbot advice must first be linked to a defined need for communication, escalation, or other follow-up. A deployed dashboard, connected registry, message, or chatbot therefore demonstrates system availability, not an action change. Threshold drift, automation bias, and plausible but unsafe generated reassurance further widen this gap [5-7]. The practical question is narrower: after a defined AI output, what action opportunity existed, who was accountable, and what happened next?

## Defining a Public Health Action End Point

We use *public health action end point* to describe a structured, AI output–specific specification of an existing proximal process or implementation end point, not a wholly new family of outcomes. It is prespecified, auditable, actor bound, time bound, and denominator based. Each analytic unit begins with a defined AI output and records whether a predefined action opportunity was completed, appropriately deferred, missed, unresolved, or lost from the denominator. Its distinctive contribution is the combination of output provenance, a named accountable actor, a defined action window, and a traceable action status. It is not model discrimination, calibration, user satisfaction, alert volume, dashboard use, message delivery, or chatbot fluency. Nor is it, by itself, proof of lower morbidity, mortality, transmission, hospitalization, or inequity.

Claim discipline means restricting the strength of a conclusion to the most distal link that has actually been measured. The end point is not a causal model; an AI output can precede an action without initiating, accelerating, changing, or causing it. Its role is to make that question observable and to prevent availability or output generation from being read as an implementation value or a population health benefit.

Justified nonaction should not become a convenient label for failure. Acceptable reasons can include an action already completed, a verified duplicate signal, a contraindication, an eligibility error, a person unreachable after a prespecified number of attempts, resource unavailability with a documented escalation pathway, or a human override with a recorded reason. Ambiguous or consequential classifications should be independently adjudicated using prespecified rules, with the adjudicator and disagreement resolution process reported.

Repeated outputs require a rule before data collection. When multiple outputs for 1 person or event fall within a prespecified episode or lockout window, they should usually be linked to 1 index action opportunity unless each output is expected to trigger a separate action. If 1 output can prompt several actions, investigators should define the primary action and report secondary actions separately. If multiple outputs contribute to 1 action, the action should not be counted repeatedly; the audit trail should preserve the outputs and the documented AI role. Where more than 1 window is clinically or operationally defensible, sensitivity analyses using alternative windows can show whether the interpretation is stable.

Textbox 1 summarizes the core end point specification and standard reporting categories.

Textbox 1. **Core specification and reporting categories for a public health action end point.**

*Core end point specification*

1. *AI output class and trigger*: the thresholded alert, score, classification, recommendation, generated message, or chatbot response that opens action consideration
2. *Action opportunity and analytic unit*: the event that makes an action eligible and the type of unit (person, alert, episode, household, facility, neighborhood, conversation, or outreach attempt)
3. *Eligible denominator and deduplication rule*: the eligible individuals or entities, applicable exclusions, and protocol for handling repeated outputs, duplicate signals, and linked actions
4. *Accountable actor and expected action*: the clinician, public health worker, program team, digital service, or agency responsible for a prespecified action
5. *Observation window*: the interval within which the action status must be recorded, justified by urgency, public health relevance, workflow capacity, and the harm of delay
6. *Action status*: completed action, predefined justified nonaction, missed action, unresolved status, or denominator loss
7. *Attribution and comparison plan*: record of the documented role of AI (initiated, accelerated, modified, supported, or no documented role) in the action and the comparator or causal design required for a causal claim
8. *Audit trail and safeguards*: source-linked time stamps, actor decisions, overrides, and model or threshold versions, with monitoring for harm, workload, privacy, equity, and drift

*Standard reporting categories*

- *Completed action* is the primary numerator for an action completion rate.
- *Predefined justified nonaction* is reported separately and is not merged with completed action in the primary numerator. A prespecified composite of appropriate disposition may be informative, but its components should remain visible.
- *Missed action*, *unresolved status*, and *denominator loss* should be reported as separate categories rather than hidden within exclusions or nonaction.

**Figure 1.**
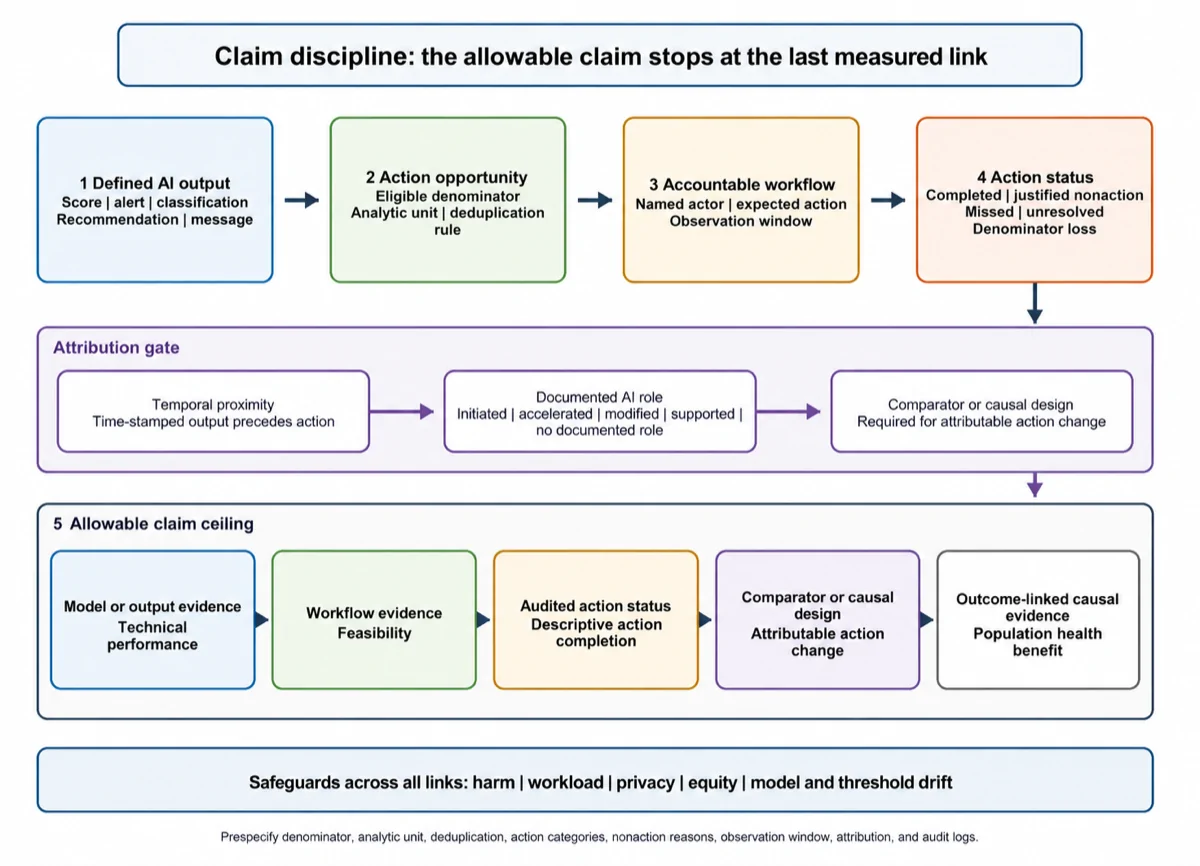
AI output–to-action pathway for public health action end points.

Figure 1 summarizes the action pathway, attribution gate, and claim ceiling that follow from the last measured link.

A defined AI output is linked to an eligible action opportunity and an accountable workflow, after which the action status is classified as completed action, predefined justified nonaction, missed action, unresolved status, or denominator loss. Time stamps and workflow records can document temporal proximity and the reported role of AI. Attributable action change requires an appropriate comparator or causal design, and a population health benefit requires outcome-linked causal evidence. The allowable claim should stop at the last measured link. Cross-cutting safeguards for harm, workload, privacy, equity, and model or threshold drift should be monitored across all links. The conceptual layout was developed with assistance from OpenAI Codex; all scientific content and the final design were reviewed and approved by the authors.

## How Public Health Action End Points Differ From Existing Evaluation Frameworks

The contribution of public health action end points is not the proposition that implementation matters. Existing implementation science, digital health reporting, quality, and AI evaluation frameworks already establish that proposition [5,8-22]. Nor do we claim that these frameworks cannot accommodate an action measure. Public health action end points instead supply a repeatable event-level specification when authors intend to claim that an AI-supported workflow changed accountable action. They can be nested within these frameworks and make explicit the provenance and denominator of the action opportunity (Table 1).

This positioning limits the framework. It is not a reporting guideline, checklist, trial design, or implementation theory. It is a structured specification of a proximal implementation or process end point. The action pathway is also not assumed to be linear; comprehension, usability, capacity, and action completion can be parallel or interacting mechanisms. The framework is useful because it requires authors to name the pathway they intend to measure instead of assuming that deployment or a downstream observation proves it.

**Table 1.** How public health action end points complement existing evaluation frameworks.

Existing framework or concept	Primary function	Contribution of an action end point when AI-supported action change is claimed
Prediction model reporting and appraisal, including TRIPOD+AI^a^ and PROBAST+AI^b^ [19,20]	Reports prediction model performance, transparency, risk of bias, and applicability	Specifies the downstream AI output, action opportunity, accountable actor, action window, and auditable action status
DECIDE-AI^c^, CONSORT-AI^d^, SPIRIT-AI^e^, and FUTURE-AI^f^ [5,16-18]	Guides early evaluation, trial reporting, protocol transparency, and trustworthy AI	Defines a public health program-level denominator and a concrete action or nonaction record after the AI output
iCHECK-DH^g^ and digital health implementation reporting [11]	Improves completeness and reproducibility of implementation reporting	Operationalizes 1 specific signal-to-action event for measurement, audit, and claim alignment
RE-AIM^h^ and Proctor implementation outcomes [8,9]	Evaluates reach, adoption, implementation, maintenance, acceptability, feasibility, fidelity, cost, penetration, and sustainability	Adds AI output provenance and event-level accountability to a broader implementation evaluation
CFIR^i^ and sociotechnical models [21,23]	Explain contextual determinants and interactions among people, tasks, technology, organizations, and policy	Defines the action event that can be interpreted within that context rather than treating the end point as context-free
Donabedian structure-process-outcome model [22]	Distinguishes structures, processes, and outcomes used to evaluate quality	Makes the AI trigger, actor, action state, and observation window explicit within an AI-mediated process link

a TRIPOD+AI: Transparent Reporting of a Multivariable Prediction Model for Individual Prognosis or Diagnosis–Artificial Intelligence.

b PROBAST+AI: Prediction Model Risk of Bias Assessment Tool–Artificial Intelligence.

c DECIDE-AI: Developmental and Exploratory Clinical Investigations of Decision Support Systems Driven by Artificial Intelligence.

d CONSORT-AI: Consolidated Standards of Reporting Trials–Artificial Intelligence.

e SPIRIT-AI: Standard Protocol Items: Recommendations for Interventional Trials–Artificial Intelligence.

f FUTURE-AI: fairness, universality, traceability, usability, robustness, and explainability in artificial intelligence.

g iCHECK-DH: Guidelines and Checklist for the Reporting on Digital Health Implementations.

h RE-AIM: reach, effectiveness, adoption, implementation, and maintenance.

i CFIR: Consolidated Framework for Implementation Research.

## Developing, Testing, and Attributing Action End Points

An action end point should be developed as a pragmatic implementation measure, not selected only because it is easy to log. Measurement quality matters in implementation research [9,24]. Before use, investigators should specify why the action is meaningful for the relevant public health program, whether the needed records can be captured reliably, and which claim the end point could support (Table 2). The table is a proposed appraisal framework, not a consensus-derived validation instrument; empirical testing is needed to establish its feasibility, reliability, and usefulness across settings.

**Table 2.** Proposed criteria for developing and appraising a public health action end point.

Domain	Question before use	Minimum evidence or reporting expectation
Meaningfulness and content validity	Does the action represent a clinically or programmatically meaningful opportunity rather than documentation alone?	State the action rationale, its link to a guideline or program objective when available, and the input of relevant operational, clinical, and community stakeholders
Feasibility and workflow sensitivity	Can the responsible actor reasonably complete and document the action in the proposed window?	Describe workflow capacity, data source, missingness, and pilot or feasibility evidence when available
Reliability and auditability	Can different reviewers reconstruct and classify the action state from the same record?	Retain source-linked time stamps and reasons; independently adjudicate ambiguous or consequential classifications and report the procedure
Attribution	What role did the AI output have in the action, and what comparison is needed for the intended claim?	Predefine role labels (initiated, accelerated, modified, supported, or no documented role) and state the comparator or causal design for causal claims
Resistance to gaming	Could denominator narrowing, post hoc nonaction labels, or repeated outputs inflate apparent success?	Prespecify denominator, deduplication, threshold, action categories, nonaction reasons, and handling of unresolved cases
Safety and equity	Could action completion create harm or mask unequal reach, burden, or performance?	Prespecify harms and stratify denominator, reach, completion, and outcomes by contextually relevant groups
Outcome linkage and claim boundary	Is there evidence that the action is plausibly related to a desired public health outcome?	State whether the claim is a descriptive action completion, an attributable action change, or a population health benefit, and avoid treating the end point as a validated surrogate

Three inferences should be kept separate. First, time-stamped logs can show *temporal proximity*, indicating that an AI output preceded an action. Second, a workflow record or actor documentation can show a *documented role*, meaning the output initiated, accelerated, modified, supported, or had no documented role in that action. Third, a causal design can estimate *attributable action change*, that is, whether an action changed because the AI implementation was introduced. The first two are necessary for an auditable pathway but are not substitutes for the third. For causal attribution, studies need an appropriate comparator or counterfactual strategy, such as randomization, a controlled before-and-after design, interrupted time series, or another design that addresses secular trends, case mix, concurrent interventions, and selective follow-up.

The selected observation window should reflect clinical urgency, public health relevance, workflow capacity, and the consequences of delay. A descriptive postdeployment action rate can support an implementation description. A claim that AI improved action requires an attribution strategy; a claim of clinical or population health benefit requires outcome-linked causal evidence. For patient-facing communication, message delivery, clicks, and conversation volume are not evidence of understanding. Comprehension should use language-appropriate, literacy-sensitive measures, teach-back, or other prespecified measures [25]. Usability may be assessed separately with an instrument such as the System Usability Scale [26]. Workload can use task time, queue or message volume, or a prespecified instrument such as the NASA Task Load Index [27].

## Operationalizing Action End Points Across Digital Public Health Use Cases

The practical test is whether a manuscript can translate a weak end point into a measurable action end point. The examples in Table 3 are author-proposed illustrations, not a systematic review–derived taxonomy or a claim that every published implementation used the listed weak end point. Their purpose is to show how the denominator, action opportunity, accountable actor, window, attribution record, safeguards, and claim boundary can be made visible.

**Table 3.** Illustrative public health action end points across digital public health AI use cases.

Use case	Common but insufficient end point	Illustrative action end point and calculation	Paired monitoring and allowable claim
Outbreak surveillance	Number of alerts or retrospective detection accuracy	Thresholded alerts reviewed within 24 hours; completed verification or escalation and predefined justified nonaction are reported separately; denominator=eligible alert episodes	False alarms, team workload, delayed true events, geographic inequity; supports descriptive action completion, while attributable action change requires an appropriate comparator or causal design
Vaccination outreach	Priority list generated or messages sent	Eligible high-risk residents contacted, scheduled, or vaccinated within 30 days, with each action reported separately and the documented AI role retained	Inequitable reach, digital exclusion, language barriers, resource diversion; supports descriptive action completion, while attributable action change requires an appropriate comparator or causal design
Chronic disease registry	Risk score assigned	Guideline-linked medication review, referral, or follow-up completed within a defined window after an AI flag; denominator=eligible flagged patients or episodes	Primary care workload, missed low-access groups, overreferral; supports descriptive action completion, while attributable action change requires an appropriate comparator or causal design
Heat-health warning	Warning issued or message opened	Eligible facilities, persons, or neighborhoods receiving check-in, cooling support, welfare assessment, or mitigation within 48 hours after a warning	Overalerting, geographic inequity, unmet social needs, service capacity; supports descriptive action completion, while attributable action change requires an appropriate comparator or causal design
Chatbot triage or risk communication	Conversation volume, answer fluency, or user satisfaction	Eligible high-risk conversations escalated to a human reviewer, public health service, emergency pathway, or other urgent pathway; lower-risk source-grounded self-care support evaluated separately	False reassurance, language and literacy bias, unsafe advice, privacy risk; supports descriptive escalation completion, while a safety claim requires prespecified error or adverse-event evaluation and an appropriate design

## Scope, Governance, and Claim Boundaries

Public health action end points apply to both research implementations and production systems, but they do not collapse the governance requirements of those settings. For research, investigators should state whether human research ethics review, trial registration, data governance approval, and funder due diligence apply to the planned evaluation. For production systems, the end point complements rather than substitutes for jurisdiction-specific regulatory requirements, institutional oversight, quality management, and postdeployment monitoring. It does not determine whether a system is regulated as a medical device or the level of evidence required for authorization.

Research ethics committees or institutional review boards, public health and clinical leaders, data governance teams, and funders where relevant should be involved before implementation. The proposed workflow should include contingency actions for unsafe outputs, capacity failures, threshold or model changes, human overrides, and fallback or rollback. This is especially important where automated recommendations or generated content can affect triage, escalation, access to services, or allocation of limited resources.

Action end points should not be imposed on every early-stage AI study. Model development, external validation, silent evaluation, and usability testing can be valuable without proving action change. Nor does the framework require every system to demonstrate population health benefit immediately. It asks that the claim stop at the stage tested (ie, technical performance, feasibility, descriptive action completion, attributable action change, or outcome-linked population health benefit). A completed-action rate alone supports only a descriptive implementation statement, a causal action-change claim requires a comparator or counterfactual strategy, and outcome claims require a design that links action changes to relevant outcomes.

The end point should not reward action volume for its own sake. Low thresholds can increase unnecessary field work, testing, anxiety, stigma, overtreatment, or resource diversion, while high thresholds can miss people or communities most likely to benefit. Unequal data quality can also make a model appear accurate while excluding digitally marginalized populations [28]. Prespecification of denominators, thresholds, action categories, nonaction reasons, duplicate rules, observation windows, equity strata, and audit logs helps prevent gaming and keeps missed action, unresolved status, and denominator loss visible.

For authors and reviewers, the key reporting question is not whether an action end point is universally required. It is whether an AI-supported action-change claim is proportionate to the action pathway that has been measured. Manuscripts making such a claim should state the output, denominator, action opportunity, accountable actor, observation window, action status categories, attribution strategy, safeguards, and intended level of inference. Without these elements, an article may be reporting a model or system rather than an evaluated AI-supported public health intervention.

## Conclusions

Digital public health AI should not be judged only by whether models classify risk, generate messages, or automate surveillance. A public health action end point makes a proximal action pathway observable by detailing what opportunity followed which AI output, for whom, by whom, within what window, with what action status and documented AI role. It is a structured process or implementation end point, not a causal outcome or a surrogate for population health benefit. The allowable claim should stop at the last link that has been measured with an appropriate attribution strategy.
